# Gender Differences in the Use of ChatGPT as Generative Artificial Intelligence for Clinical Research and Decision-Making in Occupational Medicine

**DOI:** 10.3390/healthcare13121394

**Published:** 2025-06-11

**Authors:** Patricia Mashburn, Felix A. Weuthen, Nelly Otte, Hanif Krabbe, Gerardo M. Fernandez, Thomas Kraus, Julia Krabbe

**Affiliations:** 1 Institute of Occupational, Social and Environmental Medicine, Medical Faculty, RWTH Aachen University, 52074 Aachen, Germany; 2 Department of Vascular Surgery, St. Josef Hospital Bochum, Katholisches Klinikum Bochum, Medical Faculty, Ruhr University Bochum, 44791 Bochum, Germany; 3 Faculty of International Healthcare Management (Public Health), IU International University of Applied Sciences (Berlin), 10247 Berlin, Germany; 4 Institute for Prevention and Occupational Medicine of the German Social Accident Insurance, Institute of the Ruhr University Bochum (IPA), Medical Faculty, Ruhr University Bochum, 44789 Bochum, Germany

**Keywords:** ChatGPT, artificial intelligence, large language models, gender, occupational medicine

## Abstract

**Background/Objectives:** Artificial intelligence (AI) has evolved from early diagnostic expert systems to advanced generative models, such as GPT-4, which are increasingly being used in healthcare. Concerns persist regarding inaccuracies and input dependency. This study aimed to deliver initial insights into whether gender influences the interaction of medical professionals with generative AI. **Methods:** This analysis investigated gender differences in medical students’ and physicians’ interactions with ChatGPT-4 while researching occupational medicine cases in a randomized controlled study. Participants assessed cases involving asbestos-related disease, metal sulfate allergy, and berylliosis using ChatGPT. Inputs and outputs were evaluated for accuracy, confabulations, communication styles, and user satisfaction. Demographic data and self-assessments of occupational medicine knowledge before and after the tasks were also collected. **Results:** Among 27 participants (63% women, 37% men), women showed greater knowledge improvement after using ChatGPT, particularly in asbestos-related cancer identification. No significant gender differences emerged in diagnostic accuracy, reporting procedures, or satisfaction with ChatGPT. Women exhibited significantly higher self-rated competence after using the ChatGPT application, while men only showed minimal change. Input from the female participants led to more confabulations, although response accuracy remained comparable. **Conclusions:** This study offers the first real-world insights into the use of generative AI in occupational medicine, highlighting the importance of understanding user-dependent variability in AI-supported clinical practice and decision-making. These findings underscore the need for gender-sensitive AI literacy training in medical education, accommodating diverse interaction styles and strategies to mitigate AI-generated misinformation. Future research with larger and more diverse cohorts could provide deeper insights into the influence of gender, age, and experience on AI utilization in healthcare. Integrating gender-based interaction differences into AI training and applications may improve clinical performance and promote more equitable healthcare practices.

## 1. Introduction

The concept of artificial intelligence (AI) was first introduced at the Dartmouth Conference in 1956 [1]. Early medical applications of AI were limited to simple research and conceptual work. In the early 1970s and 1980s, the first medical expert systems, such as MYCIN, CASNET, and INTERNIST-I, had their beginnings. MYCIN, one of the first rule-based systems, was developed to assist physicians in diagnosing bacterial infections and providing antibiotic therapy [2]. Systems such as INTERNIST-I [3] and CASNET [4] have taken this a step further by focusing on more complex diagnostic questions, thus contributing to the development of knowledge representation and clinical reasoning tools for the medical domain. From 2010 to 2020, improvements in deep learning changed the way AI was used in healthcare applications. Regulatory agencies began to limit, regulate, and approve the clinical use of AI algorithms as entities that could improve diagnostics, outcomes, and efficiency in healthcare [5].

In recent years, generative models, including GPT-3 and GPT-4 (generative pre-trained transformers), combined with other LLMs (large language models), have begun to rapidly change the application and routine use of generative AI by internet users, especially younger users. In a medical context, generative AI is increasingly being tested and implemented into clinical routines, from facilitating medical documentation [6] to aiding in diagnostic decision-making [7] and improving patient interactions [8]. A recent umbrella review concluded that ChatGPT holds great potential as an educational and clinical support tool in healthcare, but its safe and effective use depends on strong ethical guidelines and regulatory frameworks [9]. Furthermore, it underscores the need to address issues like bias, overreliance, and trust through coordinated efforts across the healthcare sector. Thus, experts are speaking out against their unregulated use [10]. One reason for this is that the use of ChatGPT-4 can frequently result in so-called “confabulations” or “hallucinations,” where the system generates fabricated references or inaccurate information [11]. Another reason is that the results are highly dependent on the quality and precision of the user’s input [7]. Confabulations in LLMs are factually incorrect or fabricated outputs that appear fluent and plausible, making them difficult to detect without expert knowledge. These errors occur because LLMs generate responses based on statistical patterns in training data, without a built-in mechanism to verify factual accuracy [12].

In general, women and men exhibit different communication behaviors [13] that extend to their use of information and communication technology (ICT) [14]. Such differences in ICT use may imply variations in quality outcomes, particularly in specialized fields like medicine, while using generative AI systems. Furthermore, gender differences exist in language use in the context of computer-mediated communication [15,16]. Men seem to be more inclined to communicate in a direct language, with a primary focus on the content of the message. In contrast, women seem to focus on facilitating interpersonal connections and social interactions [15,16]. Recent studies have highlighted gender-based differences in the interaction with LLMs in terms of usage frequency, perceived utility, and communication style. One study found that male university students tend to use ChatGPT for longer sessions, whereas female students use it more frequently but for shorter periods [17]. Another study reported that men predominantly utilize LLMs for analytical or technical tasks, while women more often rely on them for academic writing and theoretical explanations [18]. Additionally, two studies observed that users often perceive ChatGPT as having a gender, influencing both their language and expectations [19,20]. ChatGPT is more likely to be seen as male when used for factual or problem-solving tasks and as female when providing empathetic responses [19]. Moreover, language analyses reveal subtle gender biases in both user prompts and LLM outputs [21]. It is important to examine how these differences affect the quality of decision-making in medical practice. Understanding such differences will be important in the optimization of LLM tools and the enhancement of clinical decision-making processes. Further research in this area could provide valuable insights into improving healthcare delivery and patient outcomes.

One domain that could very well benefit from the focused application of generative AI is occupational health. If its potential is utilized properly, occupational health experts will make efficient improvements in risk assessment, strategically work out plans to ensure workplace safety, and manage work-related health problems in a much more effective way. Incorporating generative AI could make data analysis easier, help identify workplace hazards early on, and create more tailored intervention plans, all of which can lead to better health outcomes for workers.

In particular, in Germany, the field of occupational medicine is characterized by significant complexity due to regulations such as the Ordinance on Occupational Diseases (BKV—Berufskrankheiten-Verordnung) [22] and various legal frameworks. This complexity often creates uncertainty among non-specialist physicians when classifying occupational diseases, as traditional research methods may not provide sufficient or clear information.

This study explores gender-specific differences in the use of ChatGPT by medical students and physicians while solving occupational lung disease cases. As large language models are increasingly integrated into clinical education, understanding how user characteristics such as gender, expertise, and communication style could affect interactions with ChatGPT is essential. Little is known about how gender influences these interactions. By analyzing real user inputs and outcomes, this study aims to provide initial insights into differential communications and outcomes. In this study, a subgroup analysis of a superordinate study [23] was conducted, in which three cases from the field of occupational medicine were designed and presented to a group of students and physicians. The participants were assigned to solve the cases using ChatGPT. In addition, the entries made by participants were recorded and analyzed for further comparison. A comparative analysis was conducted to evaluate potential differences in the use of generative LLMs between male and female participants, with a focus on variations in the quality and accuracy of output generated.

## 2. Materials and Methods

### 2.1. Study Design

**Figure 1:**
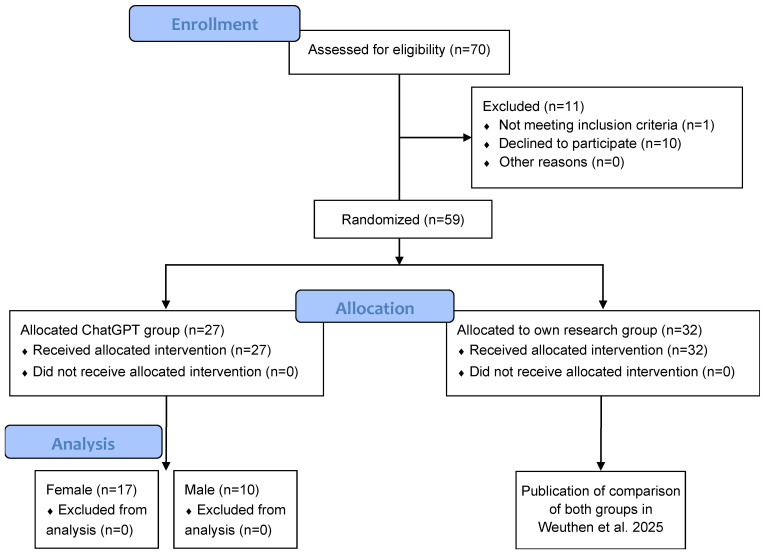
CONSORT flow diagram. This study includes a gender-stratified subgroup analysis of a superordinate study [23]; 27 participants (17 female, 10 male) were analyzed.

The current study is a subgroup analysis of a recent study comparing ChatGPT-4 and common internet research conducted by physicians and medical students [23] (Figure 1).

Using a virtual coin toss, participants were randomly assigned to one of two groups, as follows: (1) research via ChatGPT-4 or (2) research with internet tools routinely used by the participants, e.g., Google or UpToDate. Demographic data was collected, including self-reported gender, with answer options of female, male, or diverse, according to official gender entries on German passports. After the collection of self-assessments of occupational medical knowledge, three cases involving occupational lung diseases were presented, with questions that were to be answered using the assigned research method. After case processing, satisfaction with the research method, as well as self-assessment of occupational medical knowledge post-processing, was recorded.

### 2.2. Participants

The recruitment and study processes of the superordinate project are described in detail here [23]. In brief, participants were recruited through notice board announcements and personal contacts. The online study, conducted in German, required participants to be either enrolled in a medical degree program or actively practicing as a physician. Exclusion criteria were being under 18 years of age or having no medical background.

For this subgroup analysis, only participants who were assigned to the ChatGPT group were included. Two groups were then formed according to self-reported gender. There were no participants who indicated a gender other than female or male. Since this was a subgroup analysis, no matching or pairing was conducted. The groups were formed afterward, according to gender.

### 2.3. Research Settings

The English translation of the German survey is available in Table S1 and in [23]. The survey presented three cases, each based on real occupational medicine patients and modified for the study, with six questions per case. The cases were as follows:

Case 1: An outdoor worker with incidental findings of asbestos-related pleural changes on thoracic CT, leading to a suspected occupational disease report.

Case 2: A young woman working in galvanization developed a metal sulfate allergy.

Case 3: A former dental technician with recognized berylliosis.

**Figure 2:**
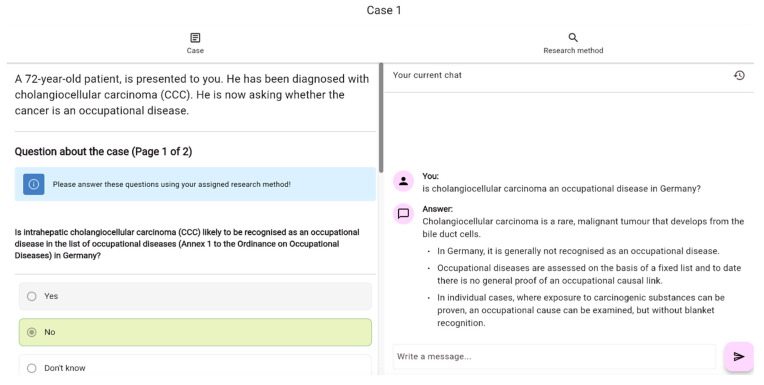
View of the ChatGPT group for solving cases. The chat window integrated OpenAI’s GPT-4 model via the Chat Completions API (application programming interface).

Each case included six questions in yes/no, multiple-choice, or free-text formats, with a “don’t know” option for each. Questions appeared three at a time alongside the case vignette on the screen. Respondents used either an integrated ChatGPT window (Figure 2) or their usual research tools, as indicated. Responses could not be altered once submitted to prevent learning effects from later questions. Copying of questions or vignettes into the chat window was disabled in order to track input accuracy and the phrasing of participants. The number of correct answers and “don’t know” answers were counted for evaluation. Comparisons were made between responses given before and after the group assignment for questions initially answered without research.

After each case, participants could choose to continue to another case or proceed to the final questions, ensuring that the concluding questions were addressed. The final questionnaire reassessed participants’ occupational medicine expertise, recorded which research tools were used, and gathered feedback on their research experience, including both positive and negative comments.

### 2.4. Research Instrument: Application

A web application was developed for the study, integrating a question-answering environment, group assignment, and case view. A chat window with a ChatGPT interface was integrated into the case view to facilitate user-friendly data collection. All data, including chat entries and responses, were stored in an SQL (structured query language) database. The application integrated OpenAI’s GPT-4 model via the Chat Completions API (application programming interface), ensuring user privacy by routing communications through private servers to prevent OpenAI from accessing participants’ IP addresses.

Participants’ input, along with previous input and responses in ongoing chats, was used as context for the LLM. The model’s responses were streamed in sections to allow for earlier viewing. The most current model at the time, GPT-4-0125-preview, was used, with a system prompt instructing it to function as an assistant for medical students and physicians investigating occupational diseases in Germany. The prompt instructed the model to ensure the accuracy of the information, to ask clarifying questions in case of ambiguity, and to provide concise answers. Default values for prompting GPT-4-Turbo were used via the Chat Completions API from OpenAI to mimic real-world usage of ChatGPT. Settings included the default temperature, t = 1.0, top_*p* = 1.0, and no max_tokens limit. The model had a max output token limit of 4096.

### 2.5. ChatGPT in- and Output

The study examined the inputs made by participants and the outputs generated by ChatGPT, including demographic data, self-assessments before and after casework, and satisfaction with ChatGPT, rated using German school grades from 1 (very good) to 6 (unsatisfactory), respectively. The number of words entered and generated was recorded, along with the nature of participants’ communication, such as whether ChatGPT was directly addressed and whether the input was provided in complete sentences or as keywords. Inputs were also analyzed for the use of adverbs (e.g., please), concretizations, personal pronouns (I, me, my), spelling mistakes, grammatical errors, and message and character counts. Notable aspects of participants’ inputs were documented. For the quantitative analysis, the number of correct answers and “don’t know” answers were counted and compared. The output analysis involved character counts, as well as the identification and assessment of confabulations and consecutive mistakes. To minimize observer bias, the analysis was conducted with the participants’ genders blinded, and were revealed only after the analysis was completed.

### 2.6. Data Analysis

The data were analyzed using GraphPad Prism version 10.2.3 (GraphPad, La Jolla, CA, USA) and SPSS version 29.0.0.0 (Statistical Package for the Social Sciences, Inc., Chicago, IL, USA). Data are presented as the number (n) with proportion in % (Table 1), the number of answers or correct answers (Tables 2–4 and 6), or as mean values with standard deviation (Tables 1–6 and 8). Group differences between genders were examined using either the Mann–Whitney U-test or the Chi^2^ test for group sizes of at least 5 individuals; otherwise, the Fisher exact test was applied (Tables 2–6 and 8) and Supplementary Table S3). Comparisons of responses before and after some questions were conducted using the Wilcoxon test (Tables 2–4). All statistical tests were two-sided, with a significance level of *p* < 0.05. Correction for multiple comparisons was conducted via the Benjamini–Hochberg procedure (false discovery rate, FDR). Multiple linear regression was used to test whether age and gender predicted self-assessment after using the ChatGPT application. The normal distribution of residuals was checked using Q-Q plots. Multicollinearity was tested using the variance inflation factor (VIF). For all independent variables, VIF values ranged between 1 and 1.8, indicating no relevant collinearity. The existence of extreme outliers could be excluded using Cook’s distance.

## 3. Results

### 3.1. Participants and Demographic Data

In the superordinate study, 70 participants were assessed for eligibility (Figure 1); 10 were excluded due to the discontinuation of the online study, and one was excluded for not indicating their medical background (thus, not meeting the inclusion criteria). The remaining 59 participants were assigned by a coin, weighted according to medical background, into one of two groups, as follows: (1) their own research or (2) ChatGPT. The comparison of these two groups is presented in [23].

**Figure 3:**
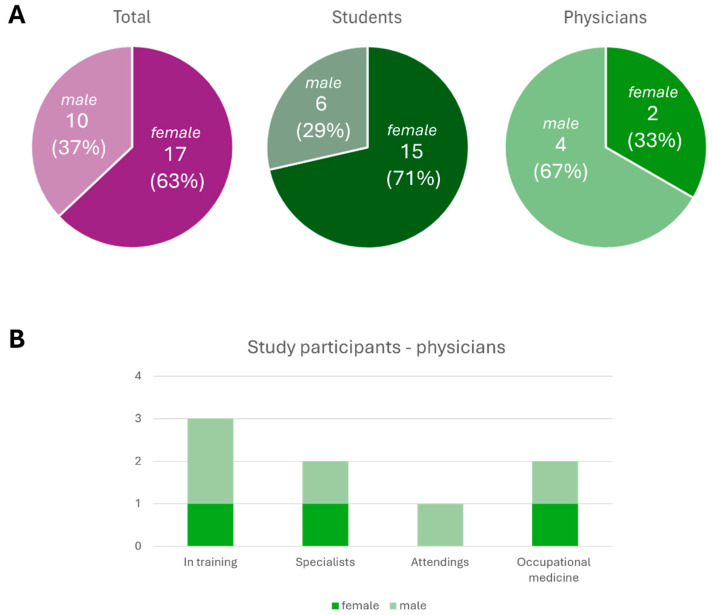
Demography of participants. (**A**) Percentages of female and male participants in total, among students and physicians. (**B**) Percentages of female and male participants among physicians in training, specialists, attendings, and occupational medicine physicians.

A total of 27 participants were placed in the ChatGPT group, consisting of 17 women (63%) and 10 men (37%) (Figure 3A). All participants chose either female or male; no ‘diverse’ indication was recorded. The demographic data can be found in Table 1; 21 of the participants were medical students, and 6 were physicians (Figure 3A). Each group included one physician with expertise in occupational medicine (Figure 3B). As the case studies progressed, the participant count decreased, leaving 15 individuals who completed all three cases. During the course, one man and six women withdrew. One woman completed the final questions without participating in the third case. The two gender groups were similar in terms of age (female 27.2 years ± 7.3 (SD), male 27.4 years ± 7.8 (SD)) and semester for the medical students (female 8.9 semester ± 1.9 (SD), male 8.7 semester ± 1.4 (SD)). In self-assessments, women rated their occupational medicine expertise higher, with an average of 3.6 ± 1.3 (SD), equivalent to “satisfactory”, compared to men, who had an average of 4.3 ± 1.3, equivalent to “sufficient” in German school grades.

### 3.2. Case Evaluation

A total of 22 participants completed case 1. There was no observed difference between genders in the number of hazardous substances listed that could lead to pleural thickening, nor in the number of materials capable of causing such an effect (Table 2). Both men and women could initially identify a similar number of cancer types caused by asbestos before using ChatGPT. However, women significantly increased their knowledge in this area after using ChatGPT compared to men. Additionally, prior to using ChatGPT, a significantly higher number of women were able to correctly state that cholangiocellular carcinoma (CCC) is not recognized as an occupational disease in Germany. After using ChatGPT, this gender difference disappeared. Also, no gender differences were found in identifying the correct occupational disease number related to asbestos-induced pleural changes according to the German occupational disease ordinance.

A total of 18 participants worked on Case 2 (Table 3). Both male and female participants showed no differences in identifying the correct diagnostic procedures or the necessary steps for officially reporting an occupational disease. After using ChatGPT, all participants were able to increase their knowledge of hazardous substances found in galvanization and the occupational fields at risk for allergic asthma development.

A total of 15 participants successfully completed the tasks of case 3. Following the use of ChatGPT, all patients demonstrated an improved ability to identify a greater number of occupations at risk for berylliosis compared to their previous knowledge (Table 4). The number of hazardous substances identified for dental technicians remained comparable between genders, as did the responses to questions concerning the procedural steps for officially reporting berylliosis as an occupational disease.

When it came to questions about sarcoidosis as an occupational disease, there was no significant difference between genders, except for one case. Before using ChatGPT, men were more likely than women to indicate that they were unsure as to whether sarcoidosis could be considered an occupational disease. However, this gender difference disappeared after ChatGPT was introduced.

### 3.3. Concluding Questions

**Figure 4:**
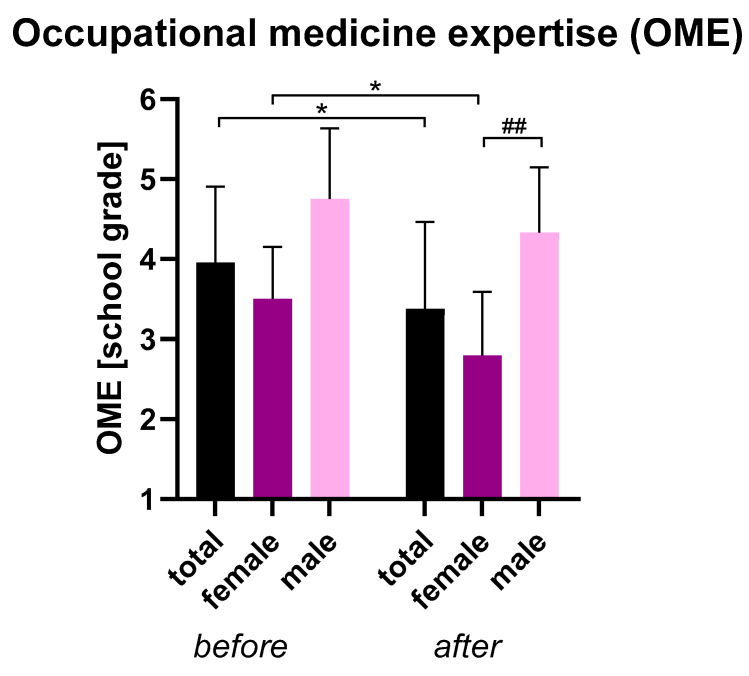
Occupational medicine expertise of participants—comparison between before and after ChatGPT use. Participants rated their self-perceived expertise according to German school grades between 1 (very good) and 6 (unsatisfactory). Mean ± SD, * *p* < 0.05 for Wilcoxon test for paired samples, ^##^ *p* < 0.01 for Mann–Whitney U test for unpaired samples.

No significant gender differences were found in satisfaction with ChatGPT, although women generally reported slightly higher satisfaction than men (Table 5). Women also initially rated their occupational health knowledge higher than men, but this difference was not statistically significant. After using ChatGPT, women showed increased self-rated competence, whereas men showed minimal change (Figure 4). Multiple linear regression was used to test whether age, gender, and baseline self-assessment predicted post-self-assessment after using the ChatGPT application. The overall regression was statistically significant (R^2^ = 0.58, F(3, 12) = 5.56, *p* = 0.01). It was found that gender significantly predicted self-assessment after ChatGPT usage (β = −1.24, *p* = 0.03), while age (β = 0.02, *p* = 0.62) and self-assessment before ChatGPT usage (β = 0.32, *p* = 0.34) did not.

### 3.4. Analysis of ChatGPT Input

The input analysis examined the frequency of certain phrases and spelling mistakes, as shown in Table 6. Although a trend was noticed where women tended to use the words “please” and personal pronouns less frequently, no significant differences were found between genders. Men, however, made nearly twice as many spelling mistakes as women. Both men and women used the phrase “one” (German: “man”) at a similar rate. Additionally, no gender differences were observed in the use of queries related to occupational diseases or in specific clarifications. Overall, the frequency of certain phrases, the number of spelling errors per person, and the correctness of grammar were comparable between men and women.

For participants, notable observations during input analysis were documented when something was outstanding, with the selection presented in Table S2. Interestingly, only women incorporated their own ideas into the cases, while both genders provided comments on short questions and keywords. Quantitative statistics were recorded as well (Table 7). Women and men had a similar number of chat messages for cases 1 and 2. On average, women wrote two more messages than men in case 3. However, this difference was not statistically significant. Women also tended to submit slightly shorter messages in terms of character count compared to men.

### 3.5. Analysis of ChatGPT Output

The output analysis involved recording ChatGPT’s character counts (Table 8) and examining the number of confabulations per case along with their impact on subsequent responses.

There were no gender differences in the character count of ChatGPT’s output, but women’s inputs tended to lead to more confabulations compared to men’s, although both genders had an equal number of consecutive errors. The difference between confabulations and consecutive mistakes was notably greater in women than in men, indicating that the increased number of confabulations did not impact answering behavior.

### 3.6. Dropout Analysis

Dropout analysis showed no significant differences between participants who dropped out during the study and participants who completed it regarding gender, age, status (student, physician), semester, and self-assessment before ChatGPT usage (Supplementary Table S3).

## 4. Discussion

This gender-stratified subgroup analysis explored the use of ChatGPT4 by female and male physicians, as well as medical students, to research occupational lung disease cases within occupational medicine. Female participants demonstrated higher baseline expertise in occupational medicine as indicated by self-assessment ratings and their ability to correctly answer knowledge-based questions without AI assistance. Although both genders benefited from ChatGPT, experiencing improvements in understanding and case-solving abilities, female participants reported greater overall satisfaction with the AI system and a more pronounced increase in self-perceived expertise following its use.

Male participants generally used more formal language, but their queries often contained grammatical or typographical errors. Conversely, female participants encountered more frequent incorrect or confabulated responses from ChatGPT. Nevertheless, these inaccuracies did not negatively impact their performance, suggesting effective identification and mitigation of misinformation.

These findings highlight significant gender-based variations in communication with ChatGPT, especially regarding self-perceived expertise. Variations in professional communication across gender and other social determinants of health have been widely documented. Gendered communication patterns in professional settings can, for example, include competence-questioning communication [16]; in male-dominated fields, assertive communication may be penalized in women [17]. Beyond gender, factors like language proficiency and cultural alignment also shape communication outcomes; individuals from minority or migrant backgrounds often face higher risks of miscommunication or reduced engagement in professional and healthcare interactions, reinforcing structural inequalities [18,19]. Regarding medical professionals, existing research consistently identifies substantial gender differences in physician self-assessment, with women often rating their abilities lower than their male counterparts, despite objectively comparable skills and qualifications [20,21,22,23,24,25]. For instance, in emergency medicine, male residents rated themselves higher than females, but external assessments showed no gender differences [21]. Similarly, female physicians rated their central venous catheterization skills lower than men, despite equal performance [22].

Several factors contribute to this phenomenon, including societal expectations that encourage women toward modesty regarding their competencies, potentially leading to systematic underestimation of their professional capabilities [26]. Moreover, female physicians frequently experience misidentification as non-physicians, which exacerbates self-doubt and negatively influences their self-perception [27]. Additionally, female physicians may internalize negative feedback more deeply, resulting in sustained adverse effects on their self-confidence, career progression, and overall job satisfaction [28].

Gender-specific communication patterns emerged in interactions with ChatGPT. Male participants generally used more formal language, incorporating polite expressions such as “please” and personal pronouns. This pattern may reflect a preference for formal or structured communication rather than perceiving the AI as a conversational partner. Such an approach is likely influenced by various social and psychological factors, shaping human interactions with AI. Polite language, including terms like “please,” often signifies respect, while the use of personal pronouns suggests engagement [29].

In the present study, participants were explicitly informed that they were interacting with a research method rather than a chatbot simulating human behavior. It is, therefore, notable that male participants maintained a formal style of interaction, while female participants did not. One possible explanation is that women, having initially rated their occupational medicine expertise higher, may have approached the AI as an equal in terms of knowledge. This perceived competence could have fostered a more confident and direct interaction. In contrast, male participants, who assessed their own expertise as lower, may have viewed themselves more as students seeking answers to relatively straightforward questions, thus adopting a more deferential communication style.

Bandura’s self-efficacy theory suggests that individuals with a stronger sense of perceived competence are more likely to demonstrate initiative and approach tasks as collaborative efforts [30]. This concept of self-efficacy has been empirically validated across diverse demographic groups regarding gender differences [31] and other socioeconomic factors like family income and supportive academic environments [32]. Self-efficacy could also be relevant in the context of AI, with users treating interactions with ChatGPT as collaborative tasks rather than purely transactional. In this study, enhanced self-efficacy among female participants facilitated confident interactions with ChatGPT, promoting deeper, more reciprocal dialogues reflective of genuine expertise. In contrast, male participants demonstrated a more deferential interaction style, typically prioritizing straightforward answers over exploratory dialogues [27].

Male queries contained more grammatical or typographical errors. Conversely, female participants encountered more frequent incorrect or confabulated responses from ChatGPT. Nevertheless, these inaccuracies did not negatively impact their performance. This suggests an ability to critically assess and filter AI-generated information. This skill may be linked to their more thorough review process. In this study, female participants employed different research strategies and approaches compared to their male counterparts. Notably, female participants more frequently requested all possible answers in multiple-choice questions, reviewed the generated output, and independently determined the correct response. In contrast, male participants were more likely to present all five answer choices to ChatGPT and rely on the AI to select the correct option. Additionally, only female participants introduced new ideas beyond the given questions, but this exploratory approach increased their exposure to AI-generated confabulations, potentially leading to the absorption of false information. These differing approaches suggest that female participants engaged in a more comprehensive review process, potentially contributing to a greater overall increase in knowledge. In contrast, male participants received only a single response per question, limiting their exposure to additional information. These gender-based patterns align with broader findings on communication differences, where women are generally more detail-focused and cautious in decision-making, while men tend to favor reliance on direct output [33,34]. Such tendencies may be further influenced by other social determinants of health, including education level, professional role, and digital literacy, which collectively shape how users communicate in general and with AI tools [35].

Notably, the female participants’ ability to fact-check ChatGPT could also stem from their existing occupational medicine expertise. With lower competency, men might have struggled to identify confabulations, potentially integrating false information into their memory and influencing case-processing outcomes.

These findings have two key implications. First, they highlight how gender-specific problem-solving strategies impact learning and the risk of absorbing misinformation, emphasizing the role of communication and decision-making styles in AI interactions. Second, they underscore the importance of AI literacy in recognizing and mitigating confabulations. Understanding these gender-associated tendencies is essential for designing AI tools that accommodate diverse learning approaches. The fact that increased exposure to confabulations did not lead to more false answers among female participants suggests that their engagement with multiple sources may serve as a cognitive safeguard against misinformation. This insight is valuable for integrating AI into education, where fostering critical evaluation skills can help prevent the spread of inaccuracies across different user groups.

### 4.1. Limitations

This subgroup analysis of the superordinate study [14] included 27 participants. However, only 15 participants completed all three cases, resulting in a very small sample size. Due to this limitation, generalizations and extrapolations to other medical specialties or physicians in general are not advisable. This subgroup analysis was conducted merely to provide initial insights into possible gender-dependent effects. In any case, the results should be interpreted with caution, as well as replicated and verified by future studies with a larger number of cases. Furthermore, particularly for case one, the number of women was higher than that of men. This relation changed over the next two cases, with an almost equal proportion of women and men for case three. However, this could have influenced the reported results. Dropout analysis showed no significant differences for participants who dropped out during the study and participants who completed it, but this does not completely rule out attrition bias. Outlier analysis revealed no relevant outliers. However, due to the small sample size and the limited recording of possible independent variables influencing outcomes, results cannot be generalized to other settings. Additionally, this study predominantly consisted of students from a certain university, introducing a potential sample and selection bias that must be considered in interpreting the findings. Nevertheless, this study represents the first investigation into the application of ChatGPT in occupational medicine.

Most existing research on ChatGPT in medicine primarily analyzes AI-generated responses to specific inquiries. To the best of our knowledge, this study is among the first to examine real user inputs from medical personnel, monitor their interactions, and assess the subsequent AI-generated outputs. Thus, despite the study’s limitations, its findings offer valuable insights, particularly for designing future studies.

Given the small sample size, complex statistical analyses were not feasible. Only significant gender differences were reported, and no comprehensive model assessing the influence of factors such as gender, age, and background could be developed. The intersectionality of these variables, which likely influences outcomes, could not be adequately examined.

Future studies should aim for larger sample sizes to enable a more robust analysis. However, recruitment proves challenging, requiring personal outreach and repeated reminders. This highlights two critical issues, as follows: participant recruitment is inherently difficult, making larger sample sizes challenging to achieve, and study samples are likely to be selective and may not fully represent the target population.

Participants completed all questionnaires independently, without the opportunity to seek clarification on instructions. While their chat interactions were recorded and reviewed, no apparent misunderstandings in case processing were observed. However, the possibility of unrecognized issues or distortions cannot be entirely ruled out. Furthermore, self-reported data on gender and medical background were not independently verified. Trans persons would have most likely stated their gender and not their gender initially assigned at birth. Nonetheless, as the primary study did not focus on gender-specific analysis and participants were unaware of such an investigation, deliberate misreporting of gender appears unlikely. Furthermore, AI literacy and experience were not characterized, nor was participants’ expertise in prompt formulation. At the beginning of the survey, participants were asked what research methods they frequently used, and none of the participants of this subgroup indicated ChatGPT. It could be concluded that participants did not routinely use ChatGPT. However, since this was not specifically asked about or evaluated, this influencing factor cannot be conclusively assessed and should be investigated in future studies.

The increasing adoption of generative AI (like ChatGPT) in healthcare introduces substantial risks related to AI-generated confabulations. Such inaccuracies pose serious implications for clinical practice, as uncritical reliance on AI-generated information can lead to clinical errors, misdiagnoses, or inappropriate treatments [36]. Aggregate effects of societal inequities are embedded in AI systems, amplifying gender and intersectional biases by replicating historical patterns of exclusion in medical training data, which risk cementing traditional roles in healthcare.

Hence, in the current state, AI should strictly serve as a supportive tool rather than a primary medical resource.

Furthermore, medical personnel should be intensively trained in the use of AI and the risks of using it in everyday clinical practice. To optimize training, integrating gender-specific insights into AI education is recommended. Leveraging diverse cognitive strengths across genders can enhance decision-making capabilities. Training should emphasize critical evaluation of AI-generated content, cross-referencing with trusted medical resources, and balancing tendencies toward overconfidence (often observed among male physicians) with thorough, cautious decision-making styles (commonly exhibited by female physicians). Employing practical training methods, such as role-playing scenarios and case studies, can facilitate collaborative learning and mutual appreciation of differing problem-solving approaches. Such training initiatives may affect end-user groups differently, as digital literacy, communication preferences, and cognitive styles vary across gender, age, and professional experience. Tailoring programs to these factors can improve engagement, reduce the risk of misuse, and promote equitable and effective adoption of AI in clinical workflows.

### 4.2. Research Implications

The finding that female participants (despite more exposure to confabulation) demonstrated higher accuracy could be explained by their higher baseline expertise in occupational medicine. This suggests that AI tools like ChatGPT may be especially effective when used by individuals with existing domain knowledge. This was also demonstrated in current research [37]. This could emphasize the need to tailor AI applications to user expertise levels. Furthermore, a user without topic-specific knowledge could pose a risk for clinical decision-making in occupational disease cases. Targeted AI training should, therefore, focus on improving confabulation detection and cross-verification strategies.

Gender disparities in technology use influence public health, particularly in family health management, patient engagement, and health education [38]. Women, often primary caregivers, frequently use digital tools for health-related tasks, while men may engage less with such platforms, limiting their role in health decision-making [39,40]. Physicians also exhibit gendered technology usage. Female physicians more frequently integrate patient-centered digital tools, enhancing communication and accessibility [41]. AI-driven health education programs that fail to account for gender preferences may struggle to engage diverse populations effectively [42].

Beyond individual user characteristics, the accessibility of LLM AI tools such as ChatGPT must also be critically examined in the context of social determinants of health. Factors such as digital literacy, access to reliable internet, language proficiency, and comfort with technology can significantly influence AI-supported learning environments [35]. These structural determinants may reinforce existing inequities if not adequately addressed. Ensuring equitable access to LLMs will be essential for harnessing their full potential in medical education and public health contexts.

In conclusion, this study highlights how gender differences, possibly linked to different communication styles, could influence the use and impact of AI tools like ChatGPT in medical education and practice. In this study, female participants, who reported higher initial expertise, engaged more collaboratively and benefited more from AI, while male participants adopted a more formal, task-oriented approach. These findings, if replicated and confirmed in other studies, could suggest the need for gender-sensitive—or at least communication style–targeted—AI training to enhance learning outcomes and promote critical evaluation skills.

Future research should examine how problem-solving strategies and self-efficacy affect AI effectiveness in clinical education. Expanding sample sizes and incorporating diverse demographics will provide deeper insights into how gender intersects with other factors in AI interactions. Longitudinal studies are needed to assess AI’s long-term impact on confidence, knowledge retention, and clinical performance.

Additionally, AI literacy training should emphasize critical thinking to mitigate risks from misinformation. Developing guidelines for AI integration in medical education and patient care will be essential. By acknowledging gender-based differences, medical education can optimize AI use, improve healthcare outcomes, and foster equitable, inclusive training for physicians.

## Figures and Tables

**Table 1:** Demographics of the participants—overall and stratified for gender. Data in n (percent) or mean value (standard deviation, SD).

n (Percent)	Total	Female	Male
n	27	17 (63.0)	10 (37.0)
Students	21	15 (88.2)	6 (88.2)
Physicians	6	2 (21.8)	4 (21.8)
In training	3	1 (33.3)	2 (66.7)
Specialists	2	1 (50.0)	1 (50.0)
Attendings	1	0 (0)	1 (100)
Occupational medicine	2	1 (50.0)	1 (50.0)
Case 1	22	14 (63.6)	8 (36.4)
Case 2	18	10 (55.6)	8 (44.4)
Case 3	15	8 (53.3)	7 (46.7)
Concluding questions	16	10 (62.5)	6 (37.5)
Mean value (SD)			
Age	27.3 (7.3)	27.2 (7.3)	27.4 (7.8)
Semester (students)	8.9 (1.7)	8.9 (1.9)	8.7 (1.4)
Self-assessment (school grades)	3.9 (1)	3.6 (1.3)	4.3 (1.3)

**Table 2:** Case 1. Gardener with asbestos-associated pleural lesions—free-text and multiple-choice questions. Participants were asked three questions, listing three answers in free text (number of responses). Free-text questions included the instruction to list hazardous substances that can cause pleural changes, materials that can induce them, and types of cancers caused by asbestos exposure. Either ChatGPT or the research method of their own choosing was used, depending on group allocation for research. Additionally, participants were asked three questions and to choose the right answers from multiple-choice questions. Via multiple-choice questions, participants were asked whether cholangiocellular carcinoma (CCC) could be officially recognized as an occupational disease (OD) in Germany, which OD number corresponded to asbestos-induced pleural changes, and whether an official OD report should be filed. The number of answers and the number of correct answers were recorded. *p* ^#^ Mann–Whitney U test for unpaired samples, *p* ^§^ Wilcoxon test for paired samples, * = *p* < 0.05, *** = *p* < 0.001. χ^2^ test for the number of correct answers, or the Fisher exact test if fewer than 5 per group.

Case 1 (n = 22)	Total	Female	Male	
Mean (SD) of number of responses				*p* ^#^
Hazardous substances with pleural changes	2.5 (0.8)	2.4 (0.9)	2.6 (0.7)	0.62
Materials causing pleural changes	1.7 (1.3)	1.6 (1.2)	1.9 (1.6)	0.72
Asbestos-related types of cancers	2.9 (0.5)	2.9 (0.3)	2.8 (0.7)	0.75
Types of cancers: before	1.8 (0.8)	1.6 (0.7)	2.0 (0.9)	0.41
Types of cancers: comparison (before vs. after, *p* ^§^)	<0.001 ***	<0.001 ***	0.12	
Number of correct answers				*p* (χ^2^)
Cholangiocellular carcinoma as an occupational disease = CCC as OD	16	9	7	0.24
CCC as OD: before	10	9	1	0.02 *
CCC as OD: comparison (before vs. after as a difference)	+6	±0	+6	-
CCC as OD: “Don’t know”	1	0	1	-
CCC as OD: “Don’t know” (before)	9	4	5	0.12
CCC as OD: “Don’t know” (comparison, before vs. after as difference)	−8	−4	−4	-
Multiple choice occupational disease: pleural changes (asbestos)	7	5	2	0.60
Occupational disease report	20	13	7	0.67

**Table 3:** Case 2. Allergy in galvanization—free-text and multiple-choice questions. Participants were asked three questions, listing three answers in free text (number of responses) or choosing the right answers from multiple-choice options. Free-text questions included the instruction to list hazardous substances workers could be exposed to in galvanization, the next steps in the diagnostic procedure for the patient in case 2, and in which occupational fields occupational asthma could occur. Additionally, participants were asked three questions and to choose the right answer from multiple-choice options. Via multiple-choice questions, participants were asked about the suspected diagnosis, the corresponding occupational disease, and whether an official occupational disease report should be filed. The number of answers and the number of correct answers were recorded. *p* ^#^ Mann–Whitney U test for unpaired samples, *p* ^§^ Wilcoxon test for paired samples, * = *p* < 0.05, ** = *p* < 0.01, *** = *p* < 0.001. χ^2^ test for the number of correct answers, or the Fisher exact test if fewer than 5 per group.

Case 2 (n = 18)	Total	Female	Male	
Mean (SD) of number of responses				*p* ^#^
Hazardous substances: galvanization	3 (0)	3 (0)	3 (0)	-
Hazardous substances: galvanization (before)	0.5 (1.0)	0.8 (1.2)	0.1 (0.4)	0.24
Hazardous substances: galvanization (comparison before vs. after, *p* ^§^)	<0.001 ***	<0.001 ***	<0.001 ***	
Diagnostic procedure	2.5 (0.5)	2.4 (0.5)	2.6 (0.5)	0.99
Occupational fields: allergic asthma	3 (0)	3 (0)	3 (0)	-
Occupational fields: allergic asthma (before)	1.8 (1.1)	1.9 (0.9)	1.6 (1.3)	0.67
Occupational fields: allergic asthma (comparison before vs. after, *p* ^§^)	<0.001 ***	0.003 **	0.03 *	
Number of correct answers				*p* (χ^2^)
Suspected diagnosis	1	5	4	1
Occupational disease	8	4	4	0.67
Occupational disease report	15	8	7	0.67

**Table 4:** Case 3. Dental technician with berylliosis—free-text and multiple-choice questions. Participants were asked two questions, listing three answers in free text (number of responses. Free-text questions included the instruction to list hazardous substances that dental technicians could be exposed to and occupational fields where occupational asthma could occur. Additionally, participants were asked four questions and to choose the right answers from multiple-choice options. Via multiple-choice questions, participants were asked whether sarcoidosis could be officially recognized as an occupational disease (OD) in Germany, which OD number corresponded to berylliosis in Germany, which diagnostic tests could be used, and whether an official occupational disease report should be filed. The number of answers and the number of correct answers were recorded. *p* ^#^ Mann–Whitney U test for unpaired samples, *p* ^§^ Wilcoxon test for paired samples, * = *p* > 0.05, ** = *p* < 0.01, *** = *p* < 0.001. For the number of correct answers, a χ^2^ test was used, or the Fisher exact test if fewer than 5 per group.

Case 3 (n = 15)	Total	Female	Male	
Mean (SD) of number of responses				*p* ^#^
Hazardous substances: dental technician	2.9 (0.5)	3 (0)	2.7 (0.8)	0.47
Occupational fields: berylliosis	2.6 (0.9)	2.6 (0.7)	2.6 (1.1)	0.99
Occupational fields: berylliosis (before)	0.2 (0.8)	0.4 (1.1)	0 (0)	0.99
Occupational fields: berylliosis (comparison before vs. after, *p* ^§^)	<0.001 ***	0.003 **	0.005 **	
Number of correct answers				*p* (χ^2^ or Fisher exact)
Sarcoidosis as an occupational disease (OD)	13	6	7	0.16
Sarcoidosis as an OD: before	5	3	2	0.31
Sarcoidosis as an OD: comparison (before vs. after as a difference)	+8	+3	+5	-
Sarcoidosis as an OD: “Don’t know”	0	0	0	-
Sarcoidosis as an OD: “Don’t know” (before)	4	0	4	0.01 *
Sarcoidosis as an OD: “Don’t know” (comparison, before vs. after as difference)	−4	0	−4	-
Occupational disease berylliosis	1	0	1	0.27
Diagnostic test	5	2	3	0.46
Occupational disease report	9	5	4	0.83

**Table 5:** Concluding questions. Survey of satisfaction and self-assessment regarding occupational medicine expertise (OME) before and after the cases. *p* ^#^ Mann–Whitney U test for unpaired samples, *p* ^§^ Wilcoxon test for paired samples, * = *p* < 0.05, ** = *p* < 0.01.

Concluding Questions (n = 16)	Total	Female	Male	
Mean value (SD) according to school grade (1–6)				*p* ^#^
Satisfaction with the research method	2.8 (1.3)	2.5 (1.2)	3.3 (1.5)	0.33
Self-assessment of occupational medicine expertise (OME): before	3.9 (0.9)	3.6 (0.7)	4.5 (0.8)	0.07
Self-assessment of OME: after	3.4 (1)	2.8 (0.8)	4.3 (0.8)	0.006 **
Self-assessment of OME: comparison (before vs. after, *p* ^§^)	0.047 *	0.02 *	0.99	

**Table 6:** Input analysis: Phrases, spelling mistakes, and grammar. *p* ^#^ Mann–Whitney U test for unpaired samples, *p* χ^2^ test, or the Fisher exact test if fewer than 5 per group.

Input Analysis (n = 22)	Total	Female	Male	
Mean value (SD)				*p* ^#^
Use of the word “please”	0.1 (0.5)	0.02 (0.2)	0.3 (0.7)	0.07
Use of “one”	0.6 (1.0)	0.6 (1.1)	0.9 (1.5)	0.70
Use of personal pronouns (I, me, my)	0.6 (1.1)	0.3 (0.6)	1.3 (1.6)	0.08
Spelling mistakes	1.4 (2.1)	1.1 (2.3)	1.9 (1.6)	0.06
Number				*p* (χ^2^ or Fisher exact)
Individual enquiry about occupational diseases	9	7	2	0.25
Concretizations	4	2	2	0.53
Use of the word “please”—persons	3	1	2	0.24
Use of “one”—persons	8	5	3	0.93
Use of personal pronouns (I, me, my)—persons	7	3	4	0.17
Spelling mistakes—persons	13	7	6	0.25
Correct grammar	5	4	1	0.39

**Table 7:** Input analysis: Chat analysis. *p* ^#^ Mann–Whitney U test for unpaired samples.

Chat Analysis (n = 22)	Total	Female	Male	
Mean value (SD)				*p* ^#^
Count of chat messages: Case1	5.8 (2.7)	5.7 (3.1)	6.0 (1.7)	0.99
Count of chat messages: Case2	6.3 (2.3)	6.6 (2.5)	6.0 (2.1)	0.71
Count of chat messages: Case3	10.5 (4.2)	11.5 (4.3)	9.3 (3.9)	0.24
Character count participants’ input: Case1	65.5 (16.3)	63.4 (13.3)	69.5 (21.3)	0.54
Character count participants’ input: Case2	61.6 (20.12)	59.5 (21.2)	64.3 (19.8)	0.63
Character count participants’ input: Case3	51.7 (27.0)	44.5 (19.2)	60.0 (33.6)	0.40
Character count ChatGPT output: Case1	1104 (267.1)	1065 (167.9)	1178 (397.9)	0.19
Character count ChatGPT output: Case2	1105 (278.3)	1149 (248.8)	1049 (319.6)	0.52
Character count ChatGPT output: Case3	1127 (260.4)	1215 (247.2)	1027 (254.9)	0.19

**Table 8:** Output analysis: Chat analysis and confabulations with their consecutive answers. *p* ^#^ Mann–Whitney U test for unpaired samples; for the number of correct answers, a χ^2^ test was used, or the Fisher exact test if fewer than 5 per group, * = *p* < 0.05.

Output Analysis (n = 22)	Total	Female	Male	
Mean value (SD)				*p* ^#^
Character count, ChatGPT output: Case1	1104 (267.1)	1065 (167.9)	1178 (397.9)	0.19
Character count, ChatGPT output: Case2	1105 (278.3)	1149 (248.8)	1049 (319.6)	0.52
Character count, ChatGPT output: Case3	1127 (260.4)	1215 (247.2)	1027 (254.9)	0.19
Confabulations	5.3 (4.1)	6.0 (4.7)	4.0 (2.7)	0.45
Consecutive mistakes	2.5 (2.1)	2.4 (2.4)	2.6 (2.1)	0.78
Confabulations vs. consecutive mistakes (*p* ^#^)	0.02 *	0.04 *	0.27	
Number				*p* (χ^2^ or Fisher exact)
Confabulations that occurred	21	13	8	0.44
Mistakes that occurred	16	9	7	0.24

## Data Availability

The data presented in this study are available upon request from the corresponding author.

## Supplementary Materials

The following supporting information can be downloaded at https://www.mdpi.com/article/10.3390/healthcare13121394/s1, Table S1: The English translation of the German survey; Table S2: Input analysis: Notes for participants regarding input.; Table S3: Dropout analysis.

## Abbreviations

The following abbreviations are used in this manuscript:

AI	Artificial intelligence
API	Application programming interface
CCC	Cholangiocellular carcinoma
ChatGPT	Chat Generative Pre-trained Transformer
LLM	large language model
OD	occupational disease
OME	occupational medicine expertise
SD	standard deviation
SQL	standard query language

## References

[1] Dzobo K., Adotey S., Thomford N.E., Dzobo W. (2020). Integrating Artificial and Human Intelligence: A Partnership for Responsible Innovation in Biomedical Engineering and Medicine. OMICS A J. Integr. Biol..

[2] Shortliffe E.H. (1976). Computer-based medical consultations: MYCIN. J. Clin. Eng..

[3] Wolfram D.A. (1995). An appraisal of INTERNIST-I. Artif. Intell. Med..

[4] Kulikowski C.A., Weiss S.M., Szolovits P. (1982). Representation of Expert Knowledge for Consultation: The CASNET and EXPERT Projects.

[5] U.S. Food & Drug administration FDA Permits Marketing of Artificial Intelligence-Based Device to Detect Certain Diabetes-Related Eye Problems. https://www.healthcare.digital/single-post/2018/04/20/fda-permits-marketing-of-artificial-intelligence-based-device-to-detect-certain-diabetes.

[6] Bongurala A.R., Save D., Virmani A., Kashyap R. (2024). Transforming Health Care With Artificial Intelligence: Redefining Medical Documentation. Mayo Clin. Proc. Digit. Heal..

[7] Cesur T., Güneş Y.C. (2024). Optimizing Diagnostic Performance of ChatGPT: The Impact of Prompt Engineering on Thoracic Radiology Cases. Cureus.

[8] Hryciw B.N., Kyeremanteng K., Fortin Z., Ghossein J. (2023). Doctor-patient interactions in the age of AI: Navigating innovation and expertise. Front. Med..

[9] Iqbal U., Tanweer A., Rahmanti A.R., Greenfield D., Lee L.T.-J., Li Y.-C.J. (2025). Impact of large language model (ChatGPT) in healthcare: An umbrella review and evidence synthesis. J. Biomed. Sci..

[10] Sallam M. (2023). ChatGPT Utility in Healthcare Education, Research, and Practice: Systematic Review on the Promising Perspectives and Valid Concerns. Healthcare.

[11] Smith A.L., Greaves F., Panch T. (2023). Hallucination or Confabulation? Neuroanatomy as metaphor in Large Language Models. PLoS Digit. Heal..

[12] Sui P., Duede E., Wu S., So R., Ku L.-W., Martins A., Srikumar V. (2024). Confabulation: The Surprising Value of Large Language Model Hallucinations. Proceedings of the 62nd Annual Meeting of the Association for Computational Linguistics (Volume 1: Long Papers).

[13] Coffman M., Marques J., Marques J. (2021). Gender and Communication: Are There Decisive Differences?. Exploring Gender at Work.

[14] Qazi A., Hasan N., Abayomi-Alli O., Hardaker G., Scherer R., Sarker Y., Kumar Paul S., Maitama J.Z. (2022). Gender differences in information and communication technology use & skills: A systematic review and meta-analysis. Educ. Inf. Technol..

[15] Tet-Mei Fung K., Chuah K.-M., Ting S.-H. (2020). Gender Differences in Computer-Mediated Communication: A Case Study on Malaysian Millennials. Humanit. Soc. Sci. Rev..

[16] Sun B., Mao H., Yin C. (2020). Male and Female Users’ Differences in Online Technology Community Based on Text Mining. Front. Psychol..

[17] Bouzar A., El Idrissi K., Ghourdou T. (2024). Gender Differences in Perceptions and Usage of ChatGPT. Int. J. Humanit. Educ. Res..

[18] Fadillah M.A., Akbar M.F. (2025). From the Gender Lens: Student Perceptions of ChatGPT in Higher Education. Adv. Mob. Learn. Educ. Res..

[19] Wong J., Kim J. (2023). ChatGPT Is More Likely to Be Perceived as Male Than Female. arXiv.

[20] Liu W. (2024). Exploring Gender Biases in Language Patterns of Human-Conversational Agent Conversations. arXiv.

[21] Mirza I., Jafari A.A., Ozcinar C., Anbarjafari G. (2025). Quantifying Gender Bias in Large Language Models Using Information-Theoretic and Statistical Analysis. Information.

[22] Federal Institute for Occupational Safety and Health Occupational Diseases. https://www.baua.de/EN/Topics/Prevention/Physical-health/Occupational-diseases/Occupational-diseases.

[23] Weuthen F.A., Otte N., Krabbe H., Kraus T., Krabbe J. (2025). Comparison of ChatGPT and Internet Research for Clinical Research and Decision-Making in Occupational Medicine: Randomized Controlled Trial. JMIR Form. Res..

[24] Briggs C.Q., Gardner D.M., Ryan A.M. (2023). Competence-Questioning Communication and Gender: Exploring Mansplaining, Ignoring, and Interruption Behaviors. J. Bus. Psychol..

[25] Rudman L.A., Glick P. (2001). Prescriptive Gender Stereotypes and Backlash Toward Agentic Women. J. Soc. Issues.

[26] Flores G. (2006). Language Barriers to Health Care in the United States. N. Engl. J. Med..

[27] Schyve P.M. (2007). Language Differences as a Barrier to Quality and Safety in Health Care: The Joint Commission Perspective. J. Gen. Intern. Med..

[28] Gude T., Finset A., Anvik T., Bærheim A., Fasmer O.B., Grimstad H., Vaglum P. (2017). Do medical students and young physicians assess reliably their self-efficacy regarding communication skills? A prospective study from end of medical school until end of internship. BMC Med. Educ..

[29] Cherney A.R., Smith A.B., Worrilow C.C., Weaver K.R., Yenser D., Macfarlan J.E., Burket G.A., Koons A.L., Melder R.J., Greenberg M.R. (2018). Emergency Medicine Resident Self-assessment of Clinical Teaching Compared to Student Evaluation Using a Previously Validated Rubric. Clin. Ther..

[30] Tzamaras H., Sinz E., Yang M., Ng P., Moore J., Miller S. (2024). Competence over confidence: Uncovering lower self-efficacy for women residents during central venous catheterization training. BMC Med. Educ..

[31] Necknig U., Wolff I., Bründl J., Kriegmair M.C., Marghawal D., Wülfing C., Burger M., May M. (2020). Gender-Specific Variations in Professional and Personal Aspects among Senior Urology Physicians at German Centers: Results of a Web-Based Survey. Urol. Int..

[32] Nomura K., Yano E., Fukui T. (2010). Gender Differences in Clinical Confidence: A Nationwide Survey of Resident Physicians in Japan. Acad. Med..

[33] Ramakrishnan A., Sambuco D., Jagsi R. (2014). Women’s Participation in the Medical Profession: Insights from Experiences in Japan, Scandinavia, Russia, and Eastern Europe. J. Women’s Health.

[34] Carnes M. (2010). Commentary: Deconstructing Gender Difference. Acad. Med..

[35] Berwick S., Calev H., Matthews A., Mukhopadhyay A., Poole B., Talan J., Hayes M.M., Smith C.C. (2021). Mistaken Identity: Frequency and Effects of Gender-Based Professional Misidentification of Resident Physicians. Acad. Med..

[36] Gentile B., Grabe S., Dolan-Pascoe B., Twenge J.M., Wells B.E., Maitino A. (2009). Gender Differences in Domain-Specific Self-Esteem: A Meta-Analysis. Rev. Gen. Psychol..

[37] Gaube S., Suresh H., Raue M., Lermer E., Koch T.K., Hudecek M.F.C., Ackery A.D., Grover S.C., Coughlin J.F., Frey D. (2023). Non-task expert physicians benefit from correct explainable AI advice when reviewing X-rays. Sci. Rep..

[38] Figueroa C.A., Luo T., Aguilera A., Lyles C.R. (2021). The need for feminist intersectionality in digital health. Lancet Digit. Health.

[39] Kim G.H., Choo E.K., Ranney M.L. (2014). Impact of Gender on Patient Preferences for Technology-Based Behavioral Interventions. West. J. Emerg. Med..

[40] Moulaei K., Moulaei R., Bahaadinbeigy K. (2023). Barriers and facilitators of using health information technologies by women: A scoping review. BMC Med. Inform. Decis. Mak..

[41] Roter D.L., Hall J.A., Aoki Y. (2002). Physician Gender Effects in Medical Communication: A meta-analytic review. JAMA.

[42] Karatsoli M., Nathanail E. (2020). Examining gender differences of social media use for activity planning and travel choices. Eur. Transp. Res. Rev..

