# Supplementary material for: Gender Differences in the Use of ChatGPT as Generative Artificial Intelligence for Clinical Research and Decision-Making in Occupational Medicine
Source: Healthcare (Basel). 2025 Jun 11;13(12):1394. doi: 10.3390/healthcare13121394 (PMC12192902; doi:10.3390/healthcare13121394)
Supplement: Supplementary file 1 [file healthcare-13-01394-s001.zip › Table S1.pdf]

Socio-demographic questionnaire:

Question	Answer options
What stage of medical training are you at?	- In medical school<br>- Physician in training<br>- Physician specialist<br>- Attending<br>- Other (free text)<br>- No specification
In which semester are you studying?<br>(In case of previous answer: In medical studies)	Answer options from semester 1 to semester 13<br>- Other (free text)<br>- No specification
What specialty are you practicing?<br>(In case of previous answer physician)	- Occupational medicine<br>- Internal medicine<br>- Pneumology<br>- Cardiology<br>- Surgery<br>- General medicine<br>- Pathology<br>- Neurology<br>- Psychiatry<br>- Radiology<br>- Anesthesiology<br>- Gynecology<br>- Urology<br>- Other (free text)<br>- Not specified
Which applications/websites do you use for medical research? (multiple choice possible)	- Amboss<br>- Thieme eRef<br>- DocCheck<br>- Via Medici<br>- Google<br>- Wikipedia<br>- UpToDate<br>- Other (free text)<br>- Not specified
Please enter your age in years	Free text
Please enter your gender	- Male<br>- Female<br>- Diverse<br>- Other (free text)<br>- Not specified
Is German your native language?	- Yes<br>- No<br>- No information
What is your level of German language skills? (If you answered no to the previous question)	- Fluent<br>- Advanced language skills<br>- Basic knowledge<br>- Other (free text)<br>- No information

Occupational medicine expertise questions (in advance):
Prompt: "Please answer the following questions solely from memory without using any assistance. For each question you have the option of checking "Don't know"."

Question	Answer options
First of all, please assess your current level of knowledge on the subject of occupational diseases affecting the lungs in Germany. The assessment is made in German school grades. 1 stands for very good, 6 for unsatisfactory.	- 1 (very good)<br>- 2 (good)<br>- 3 (satisfactory)<br>- 4 (sufficient)<br>- 5 (inadequate)<br>- 6 (unsatisfactory)<br>(Single Select)
Is an intrahepatic cholangiocellular carcinoma (CCC) likely to be recognized as an occupational disease in the list of occupational diseases (Annex 1 to the Ordinance on Occupational Diseases) in Germany?	- Yes<br>- No<br>- Don't know<br>(Single Select)
Which hazardous substances are employees in galvanisation most likely to be exposed to? Give up to 3 answers.	3 answer fields and don't know as an option
What are typical occupational fields of occupational disease no. 1110 according to the Ordinance on Occupational Diseases? Please give up to 3 answers.	3 answer fields and don't know as an option
Which types of cancers can be caused by asbestos? Enter up to 3 answers.	3 answer fields and don't know as an option
Which occupational activities can cause allergic asthma? Please give up to 3 answers.	3 answer fields and don't know as an option
Is sarcoidosis likely to be recognized as an occupational disease on the list of occupational diseases (Annex 1 to the Ordinance on Occupational Diseases) in Germany?	- Yes<br>- No<br>- Don't know<br>(Single Select)

Case 1:

Case description: A 72-year-old patient, is presented to you. He has been diagnosed with cholangiocellular carcinoma (CCC). He is now asking whether the cancer is an occupational disease.

Questions:

Question	Answer options
1) Is intrahepatic cholangiocellular carcinoma (CCC) likely to be recognized as an occupational disease in the list of occupational diseases (Annex 1 to the Ordinance on Occupational Diseases) in Germany?	- Yes<br>- No<br>- Don't know<br>(Single Select)
2) Thoracic CT shows broad-based pleural thickening. For exposure to which hazardous substances should you ask now? Give up to 3 answers.	3 answer fields and don't know as an option
3) Which occupational disease could be considered?	- No. 4103<br>- No. 4301<br>- No. 4203<br>- No. 4302<br>- No. 4112<br>- Don't know<br>(Multi Select)
4) The patient states that he has worked as a gardener all his life. The handling of which materials could have led to the pleural thickening specifically for the job as a gardener? Please give 3 answers.	3 answer fields and don't know as an option
5) Should a suspected occupational disease be reported here?	- Yes<br>- No<br>- Don't know<br>(Single Select)
6) Which cancers can be caused by asbestos? Give up to 3 answers.	3 answer fields and don't know as an option

Case 2:

Case description: A female patient, 28 years old, comes into your practice. She works in the galvanisation department of a company in the neighboring town. She has noticed reddish skin changes on her hands and an increasing cough with shortness of breath.

Questions:

Question	Answer options
1) Which hazardous substances does she most likely have contact with in galvanisation? Give up to 3 answers.	3 answer fields and don't know as an option
2) What is your suspected diagnosis?	Free text and don't know as an option
3) Which occupational disease could be considered?	- No. 4103<br>- No. 4301<br>- No. 4203<br>- No. 4302<br>- No. 4101<br>- Don't know<br>(Multi Select)
4) Which diagnostic steps should follow? Give up to 3 answers.	3 answer fields and don't know as an option
5) Should an occupational disease be reported here?	- Yes<br>- No<br>- Don't know<br>(Single Select)
6) What are typical occupational fields of occupational disease no. 4301 according to the Ordinance on Occupational Diseases? Please give up to 3 answers.	3 answer fields and don't know as an option

Case 3:

Case description: A female patient, 43 years old, is seeking advice from you. She was diagnosed with sarcoidosis with pulmonary fibrosis two years ago. However, she suspects that the disease is caused by her former job.

Questions:

Question	Answer options
1) Is it likely that sarcoidosis will be recognized as an occupational disease on the list of occupational diseases (Annex 1 to the Ordinance on Occupational Diseases) in Germany?	- Yes<br>- No<br>- Don't know<br>(Single Select)
2) When asked, the patent describess that she worked as a dental technician until a few years ago. What hazardous substances was she probably exposed to? Give up to 3 answers.	3 answer fields and don't know as an option
3) Which occupational disease could be considered?	- No. 1101<br>- No. 1109<br>- No. 1110<br>- No. 1105<br>- No. 1104<br>- Don't know<br>(Multi Select)
4) How can the disease be diagnosed?	- Leukocyte migration test<br>- Lymphocyte proliferation test<br>- Erythrocyte vitality test<br>- Platelet lethality test<br>- Don't know<br>(Single Select)
5) Should an occupational disease be reported here?	- Yes<br>- No<br>- Don't know<br>(Single Select)
6) What are typical occupational fields of occupational disease no. 1110 according to the Ordinance on Occupational Diseases? Please give up to 3 answers.	3 answer fields and don't know as an option

After completing all the cases, the particpants were asked to answer the following questions:

Question	Answer options
Please rate your current level of knowledge again after using the digital application on the topic of occupational diseases affecting the lungs in Germany. The rating is given in German school grades. 1 stands for very good, 6 for unsatisfactory.	- 1 (very good)<br>- 2 (good)<br>- 3 (satisfactory)<br>- 4 (sufficient)<br>- 5 (inadequate)<br>- 6 (unsatisfactory)
How satisfied are you with the use of your research method? The rating is given in German school grades. 1 stands for very good, 6 for unsatisfactory.	- 1 (very good)<br>- 2 (good)<br>- 3 (satisfactory)<br>- 4 (sufficient)<br>- 5 (inadequate)<br>- 6 (unsatisfactory)
Which search method did you use? Please list all the applications you used for your research, even if you did not specify them in advance.	Free text
What did you particularly like? (optional)	Free text
What did you not like? (optional)	Free text
