# Supplementary material for: Gender Differences in the Use of ChatGPT as Generative Artificial Intelligence for Clinical Research and Decision-Making in Occupational Medicine
Source: Healthcare (Basel). 2025 Jun 11;13(12):1394. doi: 10.3390/healthcare13121394 (PMC12192902; doi:10.3390/healthcare13121394)
Supplement: Supplementary file 1 [file healthcare-13-01394-s001.zip › Table S2.pdf]

Table S2 *Input analysis:* Notes for participants regarding input.

Input (n = 22)	
female	***male***
asks around a lot and more openly, questions about individual substances, ego reference	very concrete and short
Short, precise questions, direct follow-up	pure key points, reminiscent of Google search
Partly just keywords	Many empty enquiries
Some questions that are not relevant to the case questions	Short questions, wrong OD number entered
Short questions	Complete questions with capitalisation at the beginning and without mistakes, do not ask for the correct answer in multiple choice occupational diseases, but have them all explained.
Own ideas, e.g. case 1 silica dust	long and very precise questions, "maybe that will help you“, „Now choose between…“
Own ideas, e.g. case 1 mesothelioma	only bullet points, no questions
