# Supplementary material for: Gender Differences in the Use of ChatGPT as Generative Artificial Intelligence for Clinical Research and Decision-Making in Occupational Medicine
Source: Healthcare (Basel). 2025 Jun 11;13(12):1394. doi: 10.3390/healthcare13121394 (PMC12192902; doi:10.3390/healthcare13121394)
Supplement: Supplementary file 1 [file healthcare-13-01394-s001.zip › Table S3.pdf]

Table S3 *Dropout analysis:* Comparison of participants who dropped out during the study and participants who completed it. For the number participants regarding gender and status (student or physician) χ^2 test or Fisher exact test if less than 5 per group. p# Mann-Whitney U test for unpaired samples.

n (percent)	**Total**	***Dropout***	***Study completion***	***p (χ^2 or Fisher exact)***
**n**	**27**	**12 (44.4)**	**15 (55.6)**	
Female	17	9 (52.9)	8 (47.1)	0.50
Male	10	3 (30.0)	7 (70.0)	
Students	21	10 (47.6)	11 (52.4)	0.83
Physicians	6	2 (33.3)	4 (66.7)	
Mean value (SD)				***$p^{\#}$***
Age	27.3 (7.3)	27.4 (7.1)	27.2 (7.8)	0.94
Semester (students)	8.9 (1.7)	9.5 (2.0)	8.3 (1.4)	0.50
Self-assessment *before* (school grades)	3.9 (1)	3.7 (1.2)	4.1 (0.9)	0.50
